# Correlations between clinical parameters, blastocyst morphological classification and embryo euploidy

**DOI:** 10.5935/1518-0557.20230054

**Published:** 2024

**Authors:** Juliano Brum Scheffer, Rafaela Friche de Carvalho, Bruno Brum Scheffer, Ana Paula de Souza Aguiar, Luiza Pinheiro Pessoa, Daniel Mendez Lozano, Renato Fanchin

**Affiliations:** 1 IBRRA - Brazilian Institute of Assisted Reproduction, Belo Horizonte, Brazil; 2 School of Medicine, Tecnologico de Monterrey and Center for Reproductive Medicine CREASIS, San Pedro Monterrey, Mexico; 3 Professeur des Universites- Praticien Hospitalier en Medecine de la Reproduction, France, Hopital Foch, France

**Keywords:** age, aneuploidy, PGT-A, AMH

## Abstract

**Objective:**

The aim of the present study was to evaluate clinical and embryo parameters to predict embryo ploidy.

**Methods:**

In this retrospective analysis, we studied 838 biopsied day-5 blastocysts from 219 patients in the period from May 2021 to July 2022. All embryos were morphologically classified before biopsy and were divided into two groups according to genetic test results. Euploid embryos (299) were compared with aneuploid embryos (539) based on maternal age, anti-Mullerian hormone, antral follicle count, and embryo morphology.

**Results:**

Maternal age (36.2±3.0) of euploid embryos was lower than maternal age (37.1±2.5) of aneuploid embryos (*p*<0.0001). AMH levels were higher (3.9±1.2) in the group of euploid embryos than in the group of aneuploid embryos (3.6±1.3, *p*<0.0001). However, the AFC was not different in the group of euploid embryos (15.3±6.0) compared to the group of aneuploid embryos (14.5±5.9, *p*=0.07). The presence of aneuploidy was negatively correlated with top embryo quality (embryos 4AA and 4AB). All euploid embryos (299) were top quality versus 331 of 539 (61.49%) aneuploid embryos (*p*<0.0001).

**Conclusions:**

We found that euploid embryos were associated with lower maternal age, higher AMH levels, and higher quality embryos.

## INTRODUCTION

In some patients, clinical pregnancy rates increased when preimplantation genetic testing for aneuploidy (PGT-A) was performed (Sacchi *et al*., 2019). However, other authors have reported similar live birth rates after embryo transfer with and without PGT-A (Yan *et al*., 2021). Other authors found that euploidy correlated with living rates by embryo, not by patient (Sato *et al*., 2019). Recent data suggest that some markers such as maternal age, AMH level and embryo morphology might be associated with embryo euploidy.

Capalbo *et al*. (2014) suggested that poor quality blastocysts led to lower pregnancy rates. Irani *et al*. (2017) found a difference in ongoing pregnancy rates based on blastocyst morphology. Nazem *et al*. (2019) found that blastocyst inner-cell mass (ICM) grade was the most reliable predictor of pregnancy outcomes (Grade A ICM 55.6% x Grade C ICM 32.3%) together with the zona pellucida (OR-1.6; 99% CI 1.2-2.2) (Sato *et al*., 2019). This is consistent with other studies and provides evidence that zona pellucida and inner cell mass grade should be prioritized (Ahlström *et al*., 2013; Hill *et al*., 2013; Du *et al*., 2016).

In previous studies we demonstrated that age and AMH may be used to predict ovarian stimulation outcome. We also found that age is the best predictor of embryo quality. (Scheffer *et al*., 2017). Reig *et al*. (2020) retrospectively reviewed 8,175 single euploid ETs and demonstrated that maternal age does have a negative impact on implantation beyond ploidy status. Herlihy *et al*. (2022) published a retrospective cohort study and revealed that younger women have a higher chance of obtaining at least one euploid embryo more than older women (81% in women aged <35 years vs. 25% in women aged >42 years).

Embryo euploidy relates to pregnancy rate and to common clinical and embryo parameters. The aim of this study was to demonstrate which clinical and embryo parameters are related to embryo euploidy.

## MATERIALS AND METHODS

### Subjects

In this retrospective study, we studied 838 biopsied day-5 blastocysts from 219 patients in the period from May 2021 to July 2022 (Supplementary File). Patient age ranged from 27 to 43 years. All patients met the following inclusion criteria: i) both ovaries present; ii) no current or past diseases affecting ovaries or gonadotropin or sex steroid secretion, clearance, or excretion; iii) no current hormone therapy; iv) adequate visualization of ovaries in transvaginal ultrasound scans; and v) total number of small antral follicles (3-12 mm in diameter) between 1 and 32 follicles, including both ovaries. Endometriosis, tubal obstruction, low ovarian reserve and low sperm concentration were the main causes of infertility. Preliminary evaluation showed that at least 350 embryos had to be analyzed so that the study achieved a confidence >95%. All embryos were morphologically categorized prior to biopsy and divided into two groups according to genetic test results. Euploid embryos (299) were compared with aneuploid embryos (539) based on maternal age, AMH level, antral follicle count (AFC), and embryo morphology. All patients included in the study signed an informed consent term.

### Stimulation Protocol

rFSH (Gonal F, Merck-Serono Pharmaceuticals, Italy) was started with doses between 150 and 300 IU daily for 4 days with or without human menopausal gonadotropin (hMG) (Menopur; Ferring Pharmaceuticals, Germany). On the sixth day of ovarian stimulation, they were started on a GnRH antagonist 0.25 for 4 days (Cetrotide Merck-Serono Pharmaceuticals, Italy). Then the doses of rFSH and hMG were individually adjusted according to estradiol (E2) response and vaginal ultrasound findings.

When two or more follicles reached ≥16 to 18 mm, 250µg of recombinant human Chorionic Gonadotropin (Ovidrel, Merck-Serono Pharmaceuticals, Italy) was administered and oocyte retrieval occurred 35 to 36 hours later.

Intracytoplasmic sperm injection (ICSI) was routinely performed in all fertilization procedures, as described in the literature (Palermo *et al*., 1992). Fertilization was evident when two pronuclei were observed. Embryos were cultured until the day of PGT-A (day 5) in blastocyst medium (Cook Medical) and graded based on Gardner’s criteria for degree of expansion and hatching status (Gardner *et al*., 2000) before biopsy. All embryos were cryopreserved.

### Hormone Level Measurements and Ultrasound Scans

On day 3 of the cycle preceding stimulation, blood samples were taken from each woman to measure serum AMH levels, and transvaginal ovarian ultrasound scans were performed to measure the follicles.

The technique for the measurement of AMH was the most commonly used today. Serum levels of AMH were determined using an automated system with chemiluminescence detection (ACS-180; Bayer Diagnostics, Puteaux, France). Serum AMH levels were determined using an enzyme-linked immunosorbent assay (Roche). Intraand interassay coefficients of variation (CV) were <6 and <10% respectively, lower detection limit at 0.13ng/mL and linearity up to 21 ng/mL for AMH.

Ultrasound scans were performed using a 3.7-9.3 MHz multifrequency transvaginal probe (RIC5-9H; General Electric Medical Systems, Paris, France) by a single operator who was blinded for the results of hormone assays. The objective of ultrasound examination was to evaluate the number and size of small antral follicles. Follicles measuring 3-12 mm in mean diameter (mean of two orthogonal diameters) in both ovaries were considered. To optimize the reliability of ovarian follicular assessment, the ultrasound scanner was equipped with a tissue harmonic imaging system, which allowed improved image resolution and adequate recognition of follicular borders. Intra-analysis CV for follicular and ovarian measurements were <5%, and the lower limit of detection was 0.1 mm. To evaluate the bulk of granulosa cells in both ovaries, we calculated the mean follicle diameter (cumulative follicle diameter divided by the number of follicles measuring 3-12 mm in diameter in both ovaries) and the largest follicle diameter.

### Embryo biopsy and PGT-A

All embryos were cultured in sequential media (Cook Media) to the blastocyst stage. Approximately 3-8 trophectoderm (TE) cells were aspirated from high and medium grade embryos using a biopsy pipette (internal diameter, 30 mm) and dissected with a Zilos TK laser (Hamilton Thorne, MA). Biopsied TE cells were washed in GV HEPES medium (INGAMED) and PGT-A was performed using next-generation sequencing (NGS) based on the method described by the Beijing Genomics Institute (Tan *et al*., 2014).

For NGS, the genetic material within embryonic cells was isolated and amplified. DNA analysis was then performed via NGS to detect chromosome aneuploidies and some segmental aneuploidies (missing or extra segments of chromosomes). NGS can detect segments of chromosomes larger than 5 megabases (MB). NGS cannot distinguish between normal versus balanced embryos. The tests may not detect all forms of polyploidy, balanced structural chromosome abnormalities, or alterations smaller than 5 MB or in a heterochromatic region.

All embryos were frozen after biopsy and transferred on subsequent cycles.

### Ethical approval

Written informed consent was obtained from all participants before enrollment. The study was approved by the Ethics Committee of the Brazilian Institute of Assisted Reproduction and given certificate no. 150421/05194929.

### Statistical Analysis

Statistical analysis was performed by embryo. Measures of central tendency and variability (mean and standard error of the mean) were used in data following a normal distribution; median and interquartile ranges were used when a normal distribution could not be ascertained. Presence of a normal distribution was assessed by the Kolmogorov-Smirnov test. Unpaired data were compared using the unpaired Student’s t-test or the Mann-Whitney test, as appropriate. Paired data were compared using the Wilcoxon signed rank test. The Chi-squared and Fisher’s exact tests were used to compare between categorical variables. Minitab Statistical Software v21.1 was used in statistical analysis. A *p* value<0.05 was considered to indicate a statistically significant difference.

## RESULTS

**Figure 1:**
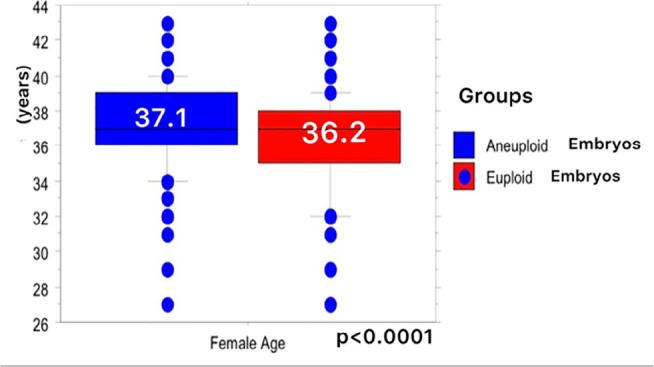
The mean maternal age of euploid embryos was lower than the mean maternal age of aneuploid embryos.

**Figure 2:**
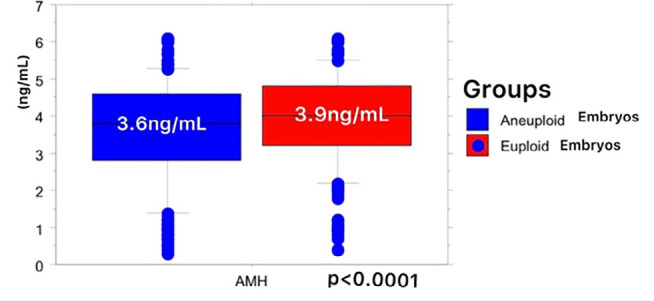
AMH levels (ng/mL) were higher in the group of euploid embryos than in the group of aneuploid embryos.

We studied 838 biopsied day-5 embryos (PGTA) from 219 patients in the period from May 2021 to July 2022. We found that the mean maternal age (36.2±3.0) of the euploid embryos was lower than the mean maternal age (37.1±2.5) of the aneuploid embryos (Table 1) (*p*<0.0001) (Figure 1). Likewise, AMH levels were higher (3.9±1.2) in the group of euploid embryos than in the group of aneuploid embryos (3.6±1.3, *p*<0.0001) (Figure 2). However, the AFC was not different in the group of euploid embryos (15.3±6.0) compared to the group of aneuploid embryos (14.5±5.9, *p*=0.07).

**Table 1:** Group characteristics.

Variable	Euploid	Aneuploid	*p*
Age (y)	36.2±3.0	37.1±2.5	<0.0001
BMI (kg/m^2^)	24.86±2.91	25.09±3.36	ns
AMHd3 (ng/mL)	3.9±1.2	3.6±1.3	<0.0001
AFCd3	15.3±6.0	14.5±5.9	ns
Total dose of rFSH (IU)	1,989±443.15	2,190±788.21	ns
Stimulation Duration (days)	10.33±1.49	10.50±1.62	ns
Follicles (total)	10.00±4.56	9.92±5.07	ns
MII Oocytes	8.279±4.43	8.06±3.24	ns
Embryos(d5) (total)	5.29±3.48	4.82±2.60	ns

We studied whether the presence of aneuploidy was associated with top embryo quality (embryos 4AA and 4AB). All euploid embryos (299) were rated as top quality versus 331 of 539 (61.49%) aneuploid embryos (*p*<0.0001).

## DISCUSSION

We examined the relationship between embryo ploidy and markers of ovarian reserve and the blastocyst morphological classification based on Gardner’s criteria. We found that age and AMH were related to embryo ploidy and top blastocyst classification (4AA and 4AB).

In other studies we published, maternal age was correlated with embryo quality (r= -0.22, *p*=0.02). This is justified by changes in the expression of certain genes and proteins in the oocytes of older women, causing mitochondrial dysfunction and consequently decrease in oocyte and embryo quality (McReynolds *et al*., 2012). Other studies found that older female age compromised oxidative phosphorylation in follicles, causing damage to mitochondrial DNA, lower oocyte quality, and embryo genetic alterations (Agarwal *et al*., 2005; Twisk *et al*., 2006; Cheng *et al*., 2009; Eichenlaub-Ritter *et al*., 2011).

In a cohort retrospective study with 225 women, Jaswa *et al*. (2021) found that euploid rates were lower among women with vs. without diminished ovarian reserve (DOR) (29.0% vs. 44.9%). The authors observed that embryos from women with DOR were more likely to be aneuploid than the ones from women without DOR after adjustment for age. Other studies support an association between low ovarian reserve and embryo quality, aneuploidy, and risk of miscarriage (Silberstein *et al*., 2006; Rosen *et al*., 2011; Tarasconi *et al*., 2017).

Our study also found a relationship between serum AMH and embryo euploidy. AMH is a marker of ovarian reserve related to the number of eggs and some studies demonstrated an association with oocyte quality. However, the use of AMH as a marker of oocyte quality is doubtful. Studies have prospectively examined the association between AMH and miscarriage among women with no history of infertility (Hagen *et al*., 2012; Zarek *et al*., 2015). In a prospective cohort study, Lyttle Schumacher *et al*. (2018) analyzed 533 women between 30 and 44 years of age and confirmed that risk of miscarriage decreased as AMH increased (risk ratio per unit increase in natural log of AMH 1⁄4 0.83 [CI], 0.73, 0.94) and women with severely diminished ovarian reserve (AMH ≤0.4ng/mL) miscarried at over twice the rate of women with AMH >1ng/mL (hazard ratio, 2.3; 95% CI, 1.3, 4.3).

In contrast, two studies did not demonstrate an association between AMH and increased miscarriage occurrence (Zarek *et al*., 2015; Pereira *et al*., 2016). Pipari *et al*. (2021) found that although a higher number of biopsied embryos were found to have higher AMH levels (*p*=0.017), a lower rate of biopsied blastocysts per metaphase II (*p*=0.019) and per fertilized oocyte (*p*=0.023) was seen in the group with high AMH levels. The authors reported an association between a greater number of euploid embryos and AMH levels (*p*=0.031); however, the rate of aneuploid embryos per metaphase II or per fertilized oocyte was not significantly different between groups (Pipari *et al*., 2021). One of the explanations for the association between AMH and embryo genetic alteration is its direct relationship with female age. The main cause of miscarriage is fetal anomalies common in older women who have lower levels of AMH.

Embryo morphological classifications have been used for years as parameters to predict pregnancy. With the advent of preimplantation genetic testing for aneuploidy (PGT-A), some assisted reproduction centers have used it to select embryos for transfer, often in association with morphological evaluation. However, PGT-A is expensive and not available in every country. Therefore, one of the objectives of this study was to demonstrate the relationship between top morphology embryos and ploidy. Some studies have described a relationship between morphological evaluation of embryo aneuploidy (Alfarawati *et al*., 2011; Capalbo *et al*., 2014; Minasi *et al*., 2016). Although this was not the objective of the study, the authors found that among the most relevant morphological characteristics, euploidy was related to the degree of expansion and hatching status.

In a study enrolling 107 patients, Gardner *et al*. (2000) showed that when blastocysts were from the two top-scoring classes, 4AA or 4AB (64% of patients), implantation and pregnancy rates were 70% and 87%, respectively. In contrast, when only low-scoring blastocysts were available for transfer (15% of patients), implantation and pregnancy rates were 28% and 44%. As previously reported (Almagor *et al*., 2016; Richter *et al*., 2001; Subira *et al*., 2016; Ahlström *et al*., 2011), the retrospective cohort study with 11,348 biopsied blastocysts by Zhan *et al*. (2020) showed that morphological classification predicted embryo ploidy and implantation rate.

## CONCLUSION

In the present study, we found that euploid embryos were associated with lower female age, higher AMH levels, and higher quality embryos. Further studies must be carried out to confirm and improve the accuracy of our results.
